# Endovascular Endocarditis Within the Superior Vena Cava of a Patient With a Tunneled Catheter for Hemodialysis

**DOI:** 10.7759/cureus.23027

**Published:** 2022-03-10

**Authors:** Richard Liang, Ian Landry

**Affiliations:** 1 Medicine, New York Institute of Technology College of Osteopathic Medicine, Glen Head, USA; 2 Medicine, Icahn School of Medicine at Mt Sinai/NYC Health + Hospitals/Queens, Jamaica, USA

**Keywords:** dialysis, end-stage renal disease, septicemia, permacath infection, endovascular endocarditis

## Abstract

Tunneled hemodialysis catheters, such as permacaths, are frequently used for vascular access in end-stage renal disease (ESRD) patients. The use of these catheters is associated with bloodstream infections, thromboses, and infective endocarditis. While valvular endocarditis is a more common entity, non-valvular endovascular endocarditis is less commonly reported in the literature. Fibrin sheaths which form along the catheter may act as niduses for infection, which can then seed the surrounding tissues. We present a case of infective endovascular endocarditis originating from an infected fibrin sheath in the superior vena cava of an ESRD patient.

## Introduction

Tunneled hemodialysis catheters, such as permacaths, are frequently used for vascular access in patients with end-stage renal disease (ESRD) who have not matured their arteriovenous (AV) fistula or are poor surgical candidates for the procedure [1,2]. The use of these catheters is associated with complications such as thrombosis, intravascular catheter-related bloodstream infections, infective endocarditis and local entry site infection [3]. These complications cause significant morbidity and mortality in an already vulnerable patient population [3].

A fibrin sheath may form when inserting an intravascular catheter [4]. During the catheter insertion process, injury to the endothelium may cause a pericatheter thrombus to form [4]. This thrombus may create an endothelial layer indistinguishable from the vein within 30 days and may remain intact within the vein after the removal of the catheter [4]. There is minimal research about the significance of fibrin sheaths in the setting of bacteremia but it is hypothesized to serve as a nidus for infection [5].

Cases of infective endocarditis originating from an infected fibrin sheath in the superior vena cava (SVC) have been described in the literature [4]. However, non-valvular endocarditis may be missed in transthoracic echocardiograms (TTE) and may only be detected on transesophageal echocardiograms (TEE) that undergo careful inspection protocols [6]. ESRD patients often present without fever in cases of endocarditis due to uremia impaired cellular host defenses, therefore, a low threshold for TEE should be considered in these patients [7,8]. Currently, there are no clear guidelines on the management of infected endovascular fibrin sheaths [4]. We report a case of SVC endovascular endocarditis in the setting of methicillin-sensitive *Staphylococcus aureus* (MSSA) bacteremia secondary to cholecystitis in an ESRD patient.

## Case presentation

A 48-year-old male with a significant past medical history of ESRD on hemodialysis via a right-sided permacath in the internal jugular vein which was placed three weeks prior to presentation, non-obstructive coronary artery disease, poorly controlled hypertension and non-insulin-dependent diabetes initially presented to the surgical service after complaining of four days of right upper quadrant pain radiating to the back associated with non-bloody, non-bilious vomiting and diarrhea. On physical examination, he was febrile to 101.2F, tachypneic at 20 breaths per minute, and hypertensive with a blood pressure of 163/91 mm Hg. Oral mucosa were dry and abdominal exam was significant for right upper quadrant and right-sided costovertebral angle/flank tenderness. The initial white blood cell count was 9,000/mcL and initial imaging was significant for a stone in the neck of the gallbladder with associated wall thickening. He was started on empiric therapy with piperacillin-tazobactam and vancomycin and admitted to the surgical service for the management of sepsis from cholecystitis. Plans were made for surgical intervention. During the initial course, he developed chest pain and underwent cardiology evaluation which determined that he was a poor surgical candidate, and therefore, underwent percutaneous cholecystostomy tube placement instead of a cholecystectomy. The cholecystostomy catheter drained copious bilious fluid. Initial blood cultures returned positive for methicillin-sensitive *Staphylococcus aureus* (MSSA) and he was transferred to the medicine service for further management.

**Figure 1:**
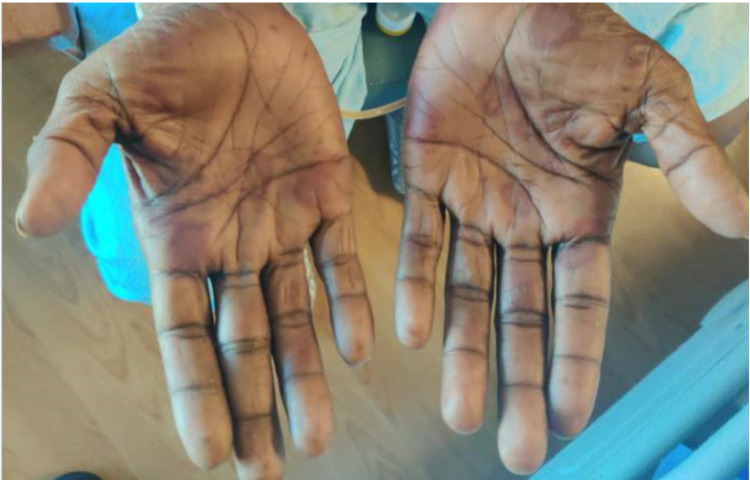
Scattered red macules on the palmar surfaces of the hands bilaterally concerning for Janeway lesions or Osler’s nodes

**Figure 2:**
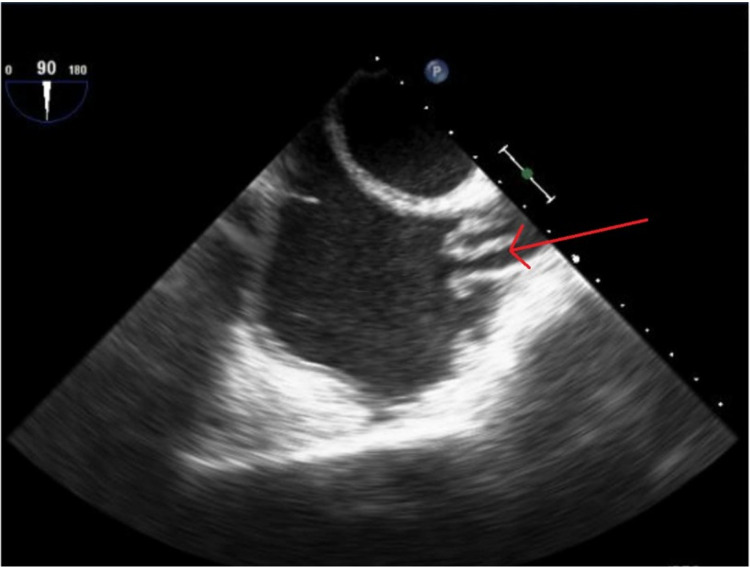
Endovascular thrombotic cast with mobile elements roughly 1.1 x 2.7 cm noted in the SVC during a TEE SVC: superior vena cava; TEE: transesophageal echocardiogram

**Figure 3:**
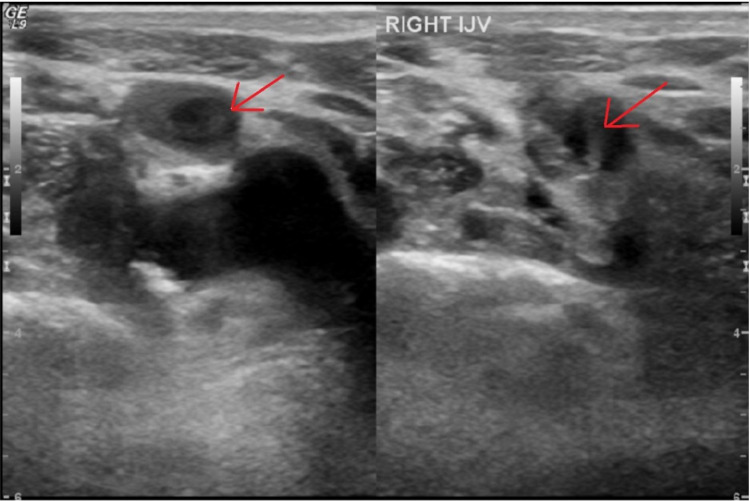
Right upper extremity duplex showing a filling defect within the right internal jugular vein

Initial transthoracic echocardiogram (TTE) was significant for preserved ejection fraction (60%) with reduced right ventricular systolic function and significant dilatation. Infectious Disease consult recommended change of antibiotics to ertapenem, removal of permacath, and evaluation for infective endocarditis. At this time, the physical exam was significant for episodes of hypoxia on room air with scattered red macules on the palmar surfaces of bilateral hands which was concerning for Janeway lesions or Osler’s nodes (Figure 1). Permacath was removed on Day 8 and subsequent transesophageal echocardiogram (TEE) on Day 9 showed no valvular vegetations but was significant for endovascular thrombotic cast with mobile elements roughly 1.1 x 2.7 cm (Figure 2). Nafcillin was started according to blood culture sensitivities and negative blood cultures were subsequently obtained on three separate days. Vascular Surgery was consulted for an opinion on the possibility of removal of the endovascular vegetated cast. Imaging of the right upper extremity with a concentration on the vasculature found a patent SVC with a new finding of a filling defect within the right internal jugular (RIJ) vein (Figure 3). Heparin drip was started for treatment of venous thromboembolism but was complicated by supratherapeutic active partial thromboplastin times in the setting of ESRD and mucosal bleeding developed from the bilateral nares, newly placed right-sided femoral Shiley catheter, and penis. 

**Figure 4:**
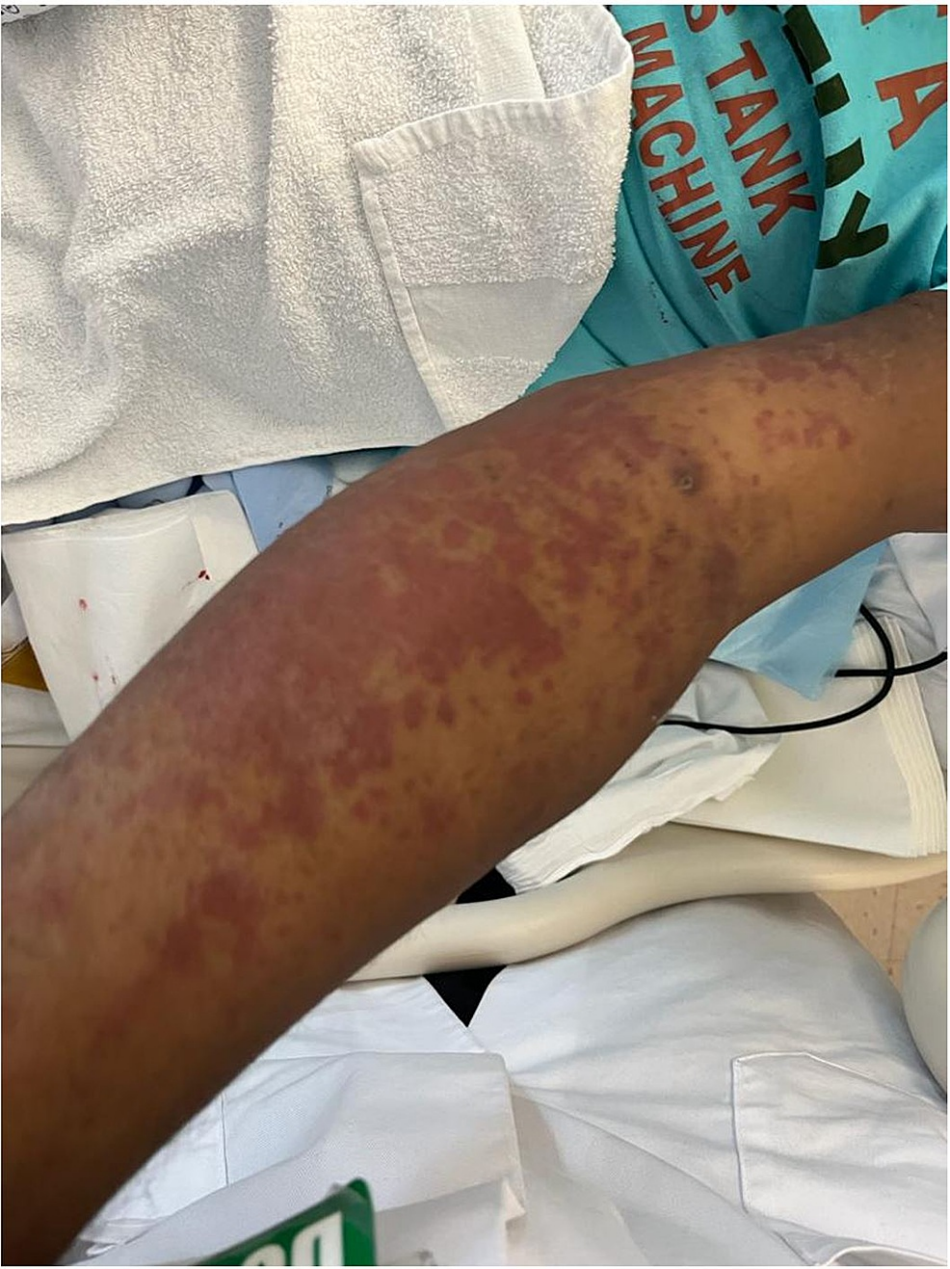
Palpable purpura on the flexor surfaces of the upper extremities concerning for delayed onset nafcillin allergy

The patient’s course was complicated by the finding of a penile lesion on the anterior surface of the foreskin which was ulcerated in nature and spread to the lateral and posterior surfaces. These lesions did not involve the urethra, scrotum or perineum. The skin began to slough and was treated by Urology. Testing for herpes simplex by polymerase chain reaction (PCR) was negative, however, empiric coverage with valacyclovir was started. During this timeframe, the patient’s mental status began to severely deteriorate and he became belligerent and agitated with staff and family members. Palpable purpura developed on the bilateral flexor surfaces of the upper extremities which was concerning for a delayed onset of nafcillin allergy (Figure 4). Empiric treatment for herpes simplex virus (HSV) encephalitis was started with intravenous valacyclovir and the decision was made to transfer the patient to an outside tertiary care facility for a higher level of care and expertise. 

**Figure 5:**
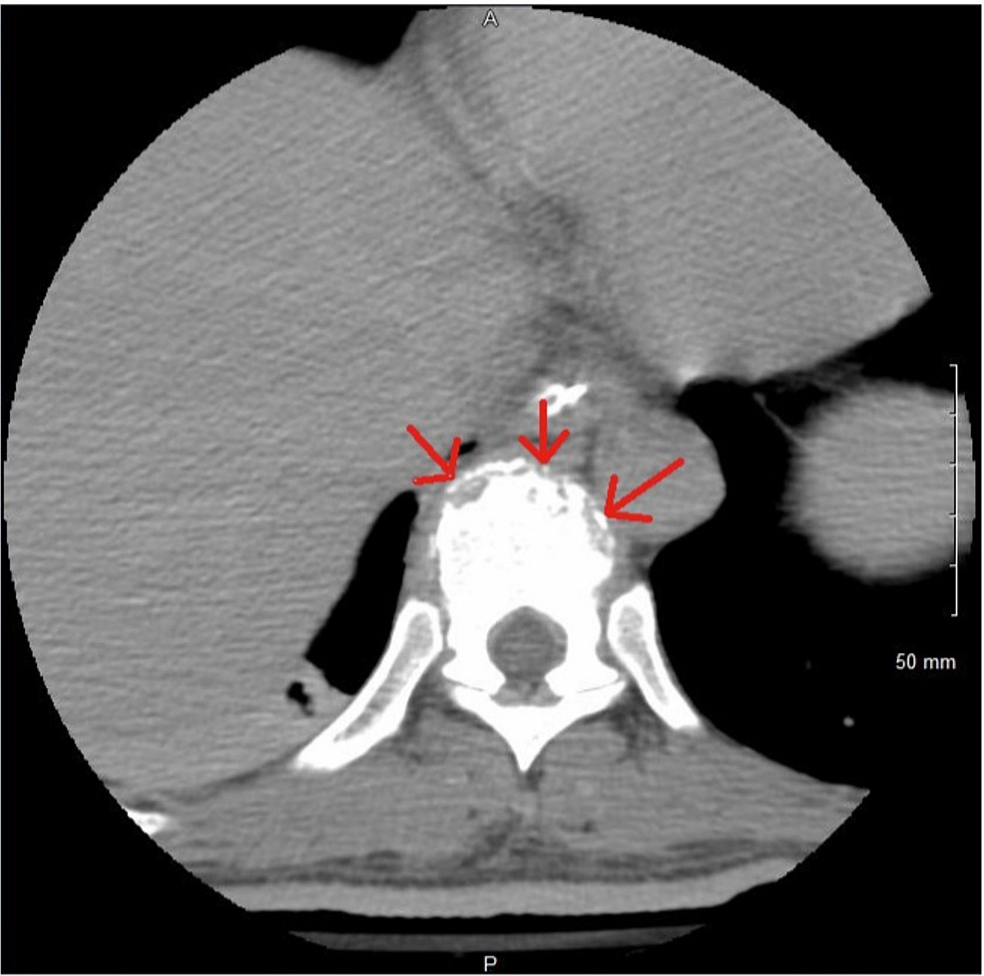
CT spine showing endplate osseous erosions around T10 suggesting vertebral osteomyelitis

**Figure 6:**
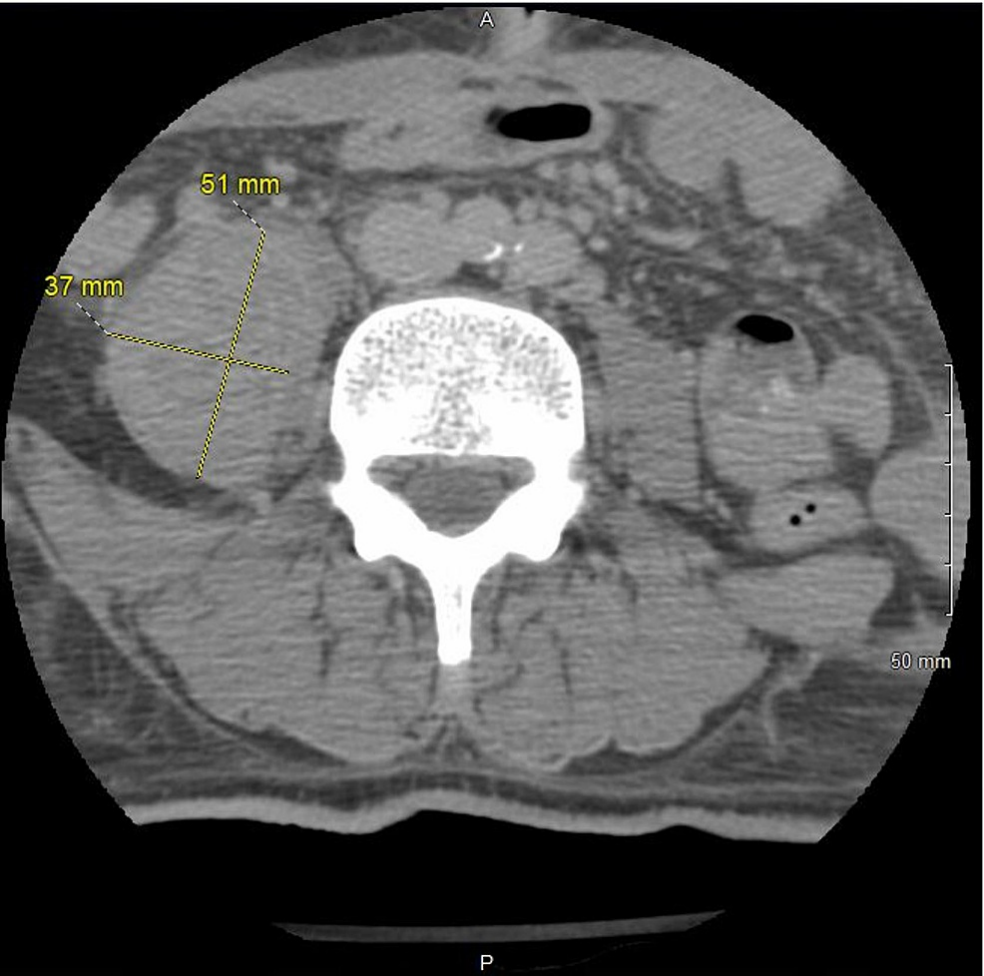
CT spine showing 3.7 cm x 5.1 cm right iliopsoas retroperitoneal hematoma versus abscess

On arrival to the outside hospital, the patient remained significantly altered in mental status with fixed, unresponsive pupils, intermittent myoclonus of the right upper extremity, and an up-going Babinski sign. A non-contrast CT of the head was negative for ischemic or hemorrhagic stroke. A bilateral upper extremity venous duplex was completed on Day 7 of his hospital stay which confirmed the resolution of the RIJ/SVC thrombus. His course was complicated by hypotensive episodes during hemodialysis which required intubation and mechanical ventilation. Vasopressors were started to maintain adequate blood pressure and video electroencephalogram (vEEG) was negative for seizure activity. CT spine showed signs of osteomyelitis in C5-C6, T-10 and L5-S1, as well as a resolving hematoma vs abscess in the right iliopsoas muscle (Figure 5, 6). Infectious disease recommended the addition of vancomycin 15 mg/kg and neurosurgical evaluation deferred surgical intervention. After 25 days of in-patient hospitalization, the patient’s mental status greatly improved and he was discharged on cefazoline for another 8 days and ultimately diagnosed with toxic metabolic encephalopathy in the setting of critical illness.

## Discussion

Left-sided endocarditis is more common and better documented than right-sided endocarditis [6]. Right-sided endocarditis only accounts for 5% to 10% of all cases and is associated with intravenous drug use, intracardiac devices and central venous catheters, all of which are becoming more prevalent in the United States over the past 20 years [6]. Non-valvular endocarditis such as superior vena cava (SVC) endocarditis is an even more uncommon presentation and only documented in a handful of cases in the current literature [4,7,9,10]. Non-valvular endocarditis in the SVC may be undetectable when performing a TTE, as with our patient, so patients with a clinical suspicion for endocarditis should undergo a TEE for a more definitive answer, especially in cases of bacteremia [6].

In a study done by Sayani et al., 12 of 87 patients (17.3%) with tunneled hemodialysis catheters developed a serious infection [11]. Only two of these patients were successfully treated with conservative management while the other 10 patients had to have the catheter removed [11]. One patient (1.3%) died from catheter-related septicemia [11]. Estimates of fibrin sheath formation occur within roughly 50-80% of all patients with a tunneled dialysis catheter [5]. Retrospective studies by Shanaah et al. subdivided dialysis patients into three groups (1) catheter exchange with fibrin sheath, (2) catheter exchange without fibrin sheath, and (3) de novo catheter placement [5]. Once a dysfunctional catheter was exchanged, the incidence of infection among the three groups was similar and not statistically significant, indicating that if appropriately treated there are similar outcomes regardless of the fibrin sheath [5]. Evidence-based preventive tactics remain crucial in reducing the risk of serious catheter-related complications [12].

## Conclusions

Patients with ESRD commonly require vascular access with tunneled hemodialysis catheters, such as permacaths. Their use, however, comes with some risks such as bloodstream infections, thromboses, and infective endocarditis. Fibrin sheaths may develop during the placement of these catheters which may serve as a nidus for infection. The threshold for the use of TEE should remain low in patients with tunneled hemodialysis catheters and bacteremia since detecting non-valvular endocarditis (SVC), such as in our patient, is difficult with a TTE. Early detection and treatment of these atypical presentations of endocarditis can prevent morbidity and mortality. Serious catheter-related complications can be avoided by practicing evidence-based preventative tactics.
